# Non-cognitive Characteristics of Gifted Students With Learning Disabilities: An In-depth Systematic Review

**DOI:** 10.3389/fpsyg.2018.00504

**Published:** 2018-04-20

**Authors:** Else Beckmann, Alexander Minnaert

**Affiliations:** Special Needs Education and Clinical Educational Sciences, Behavioural and Social Sciences, Special Needs Education and Youth Care, University of Groningen, Groningen, Netherlands

**Keywords:** twice-exceptional, learning disabilities, giftedness, non-cognitive, potential

## Abstract

Gifted students who also have learning disabilities (G/LD) are often overlooked when students are assessed either for giftedness or specific learning disabilities. The cognitive and non-cognitive characteristics of these G/LD students are habitually discussed only briefly alongside identification and intervention issues and, beyond that, the relevance of non-cognitive characteristics is often left unconsidered. Accordingly, this study aims to conduct an in-depth review of the non-cognitive characteristics of these students for identification and intervention purposes. Detailed analysis was performed on 23 publications. High levels of negative emotions, low self-perception, and adverse interpersonal relationships, as well as high levels of motivation, coping skills and perseverance were found among these students. A common characteristic was a high degree of frustration with the academic situation. The study reveals that these students show considerably duality in their non-cognitive characteristics which requires tailored counseling skills to provide effective support for their learning needs.

## Introduction

The concept of gifted students who also have learning disabilities (G/LD students), also referred to as “twice-exceptional” students, has become widely accepted (Brody and Mills, 1997). Increasing awareness of high potential students who simultaneously struggle with academic tasks (Ruban and Reis, 2005) has paved the way to recognition of this concept. Familiarity with the G/LD concept continues to increase (Nicpon et al., 2013) and literature on G/LD students is expanding, especially with regard to addressing the topic of identification and intervention (Lovett, 2013). These G/LD students are considered “twice-exceptional” because they statistically fall into the exceptional range for their cognitive, academic, or creative abilities and potential, and also fall in the lower end of being exceptional in the learning deficit area.

Lovett and Sparks (2011) show, however, that only a very small number (~5%) of articles on this subject use empirical data. Much is written about this population based on very little empirical evidence and there is not yet much consensus on how to *define* and *identify* these students (McCoach et al., 2001; Ruban and Reis, 2005). Nonetheless, even though the definition and identification of G/LD students is not clear-cut, and research-driven empirical investigations on G/LD remain scarce (Newman and Sternberg, 2004), recommendations are already being made for the assessment and education of this population (Lovett and Sparks, 2011).

As twice-exceptional students are substantially underidentified and underserved (Barnard-Brak et al., 2015), researchers have tried to identify the characteristics that describe these students (Newman and Sternberg, 2004). To identify these students correctly, it must be clear which characteristics they possess (McCoach et al., 2001) and how characteristics from both exceptionalities interact (Reis and McCoach, 2000). The reality is that G/LD students are often overlooked when they are assessed for either giftedness or learning disabilities (Reis et al., 1995; Brody and Mills, 1997). As it is assumed that giftedness can mask G/LD students' disabilities and vice versa (Ruban and Reis, 2005), they are often not recognized and ‘considered “not good enough” for gifted programs and “too good” to qualify for accommodations’ (Silverman, 2013, p. 14). This may result in G/LD students being denied access to appropriate educational and career opportunities (Ruban and Reis, 2005). Therefore, a better understanding of the distinguishing characteristics of this population is necessary for proper identification and aligned intervention (McCoach et al., 2001; Assouline and Whiteman, 2011; Barnard-Brak et al., 2015).

In light of the above, characteristics of G/LD students lie at the heart of this study. Although interrelated, a distinction can be drawn between “cognitive” and “non-cognitive” characteristics (Heckman et al., 2006). “Cognitive characteristics” usually refer to ability and/or achievement test outcomes. The term “non-cognitive characteristics” refers to the very broad range of strategies, skills, attitudes, and behaviors which play an essential role in academic performance, but may not be captured (directly) by cognitive or achievement tests (Farrington et al., 2012). These include metacognitive skills, motivation, self-esteem, creativity and personality traits (Heckman et al., 2006; Gutman and Schoon, 2013). Less attention has been paid to these non-cognitive factors (Farrington et al., 2012). Nowadays, the importance of these “soft” skills is becoming more widely accepted. For example, research suggests that high achievement is not merely a consequence of cognitive factors such as high ability, but also non-cognitive factors which play perhaps an even more important role through factors such as motivation, dedication, and hard work (Renzulli, 1978; Schneider, 2000; Heckman et al., 2006). Accordingly, this study will focus on “non-cognitive characteristics” of G/LD students.

Characteristics of G/LD students have often been the subject of literature reviews, though usually mentioned only briefly alongside definition, identification and intervention discussions (e.g., Reis et al., 1995; Brody and Mills, 1997; Reis and McCoach, 2000; Ruban and Reis, 2005). Characteristics frequently mentioned as common to these students include advanced vocabulary use, high creativity, strong critical-thinking skills, task commitment, alongside disruptive behavior, poor motivation, low self-esteem, and unrealistic self-expectations (Reis et al., 1995; Nielsen, 2002; Newman and Sternberg, 2004). The overall findings are not clear-cut however, probably because of the large variety in talents and (areas of) weaknesses exhibited by these students. The systematic literature review by Nicpon et al. (2011) covering 20 years of research, provides a comprehensive summary of the empirical literature on twice-exceptional students. Regarding the characteristics of G/LD students, they provide a brief discussion of the students' cognitive and academic patterns and psychosocial factors. They reported relatively consistent findings in the area of verbal abilities, which are typically stronger, and the area of non-verbal abilities, which are typically weaker. However, like the conclusions drawn by Brody and Mills (1997) and Reis and McCoach (2000), they do stress that there is a broad range of variability in cognitive characteristics of these students, making it difficult to identify a general pattern. The same applies to the area of psychosocial factors in which the authors identified only one distinctive characteristic, namely a state of negative emotions probably due to negative school experiences. However, the reason that the studies mentioned above do not appear to find a common set of characteristics might also be due to the broad and unclear identification criteria being used to identify these students. Questions arise, therefore, about exactly which population these characteristics describe.

The characteristics of G/LD students are only briefly discussed in the review study by Nicpon et al. (2011), which also addresses the issue of identification and intervention, as well as characteristics for other groups (i.e., with autism spectrum disorders [G/ASD] and with attention deficit hyperactivity disorders [G/ADHD]) within the twice-exceptional population. Therefore, the first aim of this study is to investigate and present systematically a comprehensive synthesis of the existing empirical literature on non-cognitive characteristics of G/LD students. The second aim is to determine the uniqueness of this group and how characteristics from both exceptionalities come together. In so doing, the study will investigate how characteristics of G/LD students relate to characteristics of students from contrasting groups such as gifted students without disabilities and non-gifted students with disabilities. To begin with, an overview is provided below on the conceptualization and characterization of giftedness, learning disabilities, and the combination of the two.

### Giftedness (G)

Many definitions for giftedness can be found in the research literature on giftedness. A commonly shared underlying conception in these definitions is high intelligence accompanied by a rather arbitrary threshold for when an individual should be considered gifted (Ziegler and Heller, 2000). From this perspective, intelligence is regarded as a single, homogeneous construct, often referencing Spearman's g factor of general intelligence (Simonton, 2000). In contrast to this one-dimensional view, more contemporary conceptions include various domains of intelligence within multidimensional models, such as Gagné's (2000) differentiated model of giftedness and talent, and Gardner's (1987) multiple intelligences model. Another influential model is Renzulli's (1978) Three Ring Model of high ability, which describes gifted achievement as high general intelligence, task commitment and creativity. Mönks (as cited in Mönks and Mason, 2000) expanded this model to include the interdependence between giftedness and the social settings of family, peer group, and school. The definitions of giftedness generally pertain to potential for achievements which are not necessarily reflected in performance, underlining the concept of underachievement (Ziegler and Heller, 2000). From this perspective, potential for achievements can develop if there are factors in the social or wider environment which elicit and support giftedness (Mönks and Mason, 2000). When educational deprivation, family factors, physical or psychological problems, or learning problems interfere, gifted students might not be able to maximally develop their potentials (Peters et al., 2000). Despite the acceptance of multidimensional definitions of giftedness in modern research on the gifted (Mönks and Mason, 2000), it has long been the case that children were included in gifted programmes based on their performance on narrowly defined psychometric instruments (Simonton, 2000). In line with these modern definitions of giftedness, additional suitable identification measures could include evidence of academic achievement, nomination by teachers, parents or peers, and evidence of non-cognitive behaviors (Mönks et al., 2000).

In most studies, the method of identifying G/LD students comes down to the assessment of their performance on intelligence tests and comparing their IQ scores to a predetermined cut-off score for giftedness (Lovett and Sparks, 2011). Therefore, in this study giftedness refers to intelligence in the “intellectual area” as measured by an intelligence test. Since the focus of this study is on G/LD students in an academic context, giftedness in areas of sports or music, for example, are not considered.

Due to the difficulty of capturing giftedness in a single theory or conceptualization, many differences can be found between gifted students, depending on the criteria used to define them. Consequently, we are dealing with a heterogeneous group of students demonstrating high ability or manifest talent or both, in many different domains, either in one specific area or across a whole range, and representing a broad range of degrees of giftedness, from the slightly above average to the profoundly gifted (Mönks et al., 2000). There can also be a considerable degree of intra-individual variation, referring to the asynchronous development often characteristic of gifted students. Other characteristics gifted students are often claimed to possess are a keen sense of humor, rapid comprehension, insatiable curiosity, large vocabularies, perfectionism, super-sensitivity, and self-criticism (Coleman and Cross, 2000; Lens and Rand, 2000; Perleth et al., 2000). Given these characteristics, gifted students are capable of exceptional levels of achievement (Schoon, 2000). When interacting in a non-adaptive (learning) environment, however, these students are prone to psychological or socio-emotional problems (Coleman and Cross, 2000). Fear of failure due to high expectations, feelings of depression, and feeling isolated from peers are just a few examples of the problems gifted students may experience (Freeman, 2000; Yoo and Moon, 2006). Nonetheless, under optimal conditions and with tailored support the students' giftedness can allow them to cope better with difficult situations (Freeman, 2000).

### Learning disabilities (LD)

The concept of learning disabilities is first mentioned in Kirk and Bateman (1962) referring to children with average intellectual ability or above, yet who also demonstrate learning problems. A few influential definitions of learning disabilities have since appeared, one of them stemming from the Individuals with Disabilities Education Improvement Act (IDEA) of 2004^1^. There, a specific learning disability (SLD) is defined as

a disorder in one or more of the basic psychological processes involved in understanding or in using language, spoken, or written, which disorder may manifest in imperfect ability to listen, think, speak, read, write, spell, or do mathematical calculations.

Learning problems which are primarily the result of visual, hearing, or motor disabilities, of mental retardation, emotional disturbance, or of environmental, cultural, or economic disadvantage, cannot be referred to as SLDs. Although influential, Lewis et al. (2013) identify several problems relating to the practical use of this definition. For instance, it is not clear how marked an imperfect ability to learn should be before it can be labeled a SLD. This can lead to broad variability in the severity of learning problems in children diagnosed with SLD.

In his review of definitions of learning disabilities, Swanson (1991) discusses three assumptions about learning disabilities underlying many operational definitions. The first assumption is the concept of “specificity,” referring to a learning problem as confined to a limited number of cognitive or academic domains. Another assumption concerns the “IQ-achievement discrepancy,” whereby children who are learning-disabled evidence a gap between potential and actual achievement. The third assumption, “exclusion,” refers to the separation of learning disabilities from other general handicapping conditions, and the ruling out of alternative explanations accounting for depressed achievement scores. Although critically important in the operationalization and identification of learning disabilities, these assumptions have also attracted criticism, especially regarding the use of the discrepancy formula (for a critical discussion see Restori et al., 2009).

No specific criteria were formulated for learning disabilities for this study because of the wide range of implicit identification methods used to identify learning disabilities in G/LD students (see Lovett and Sparks, 2011). The etiological component of IDEA's definition of SLD falls beyond the scope of this study, with the exception of comorbidity with other disorders which might bring unwarranted confounding effects to the fore (i.e., the assumption of exclusion). This study will focus on the second part of the definition of SLD: that participants should manifest difficulty in one or more of the mentioned academic skills. Classification is not considered a necessity, and studies were included so long as the authors stated that the participants exhibited persistent learning problems or difficulties.

LD students show a broad range of interindividual and intra-individual variation. There are many differences between these students in terms of the areas in which they experience difficulty, but also notable differences in the ability profile of the same individual, exhibiting different achievement levels in different subjects (i.e., the assumption of specificity: Hallahan and Kauffman, 2006). At the same time, commonalities also exist in the LD population. Children diagnosed with learning disabilities are predominantly boys (Morrison and Cosden, 1997), they may exhibit perceptual and coordination problems, and Attention Deficit Hyperactivity Disorder (ADHD) is not an uncommon diagnosis in these students (Hallahan and Kauffman, 2006). In terms of cognitive problems, LD students are often characterized as having problems with memory, especially short-term memory and working memory (Siegel and Linder, 1984; Swanson, 1993; Trainin and Swanson, 2005). Regarding non-cognitive characteristics, LD students often lack metacognitive skills, as reflected in the difficulty they experience in planning and monitoring their own learning processes (Swanson et al., 1993; Hallahan and Kauffman, 2006). They are also at greater risk of developing socio-emotional problems than their non-disabled peers, which can result in low self-concept, anxiety, depression, peer rejection, external locus of control, and learned helplessness (Bender and Wall, 1994; Morrison and Cosden, 1997; Elbaum and Vaughn, 2003; Hallahan and Kauffman, 2006; Galway and Metsala, 2011).

### Giftedness/learning disabilities (G/LD)

Traditionally, definitions of G/LD merely include giftedness and learning disabilities as a conjunction of two discreet entities instead of a complex merging of concepts. Lovett and Lewandowski (2006) criticized the use of these separate views and advocated a clear and comprehensive definition which can guide assessment and interventions for G/LD students. Recently, Reis et al. (2014) addressed this need by proposing an operational definition of the umbrella term of twice-exceptional learners (e.g., SLD, ADHD, ASD, and EBD), stated as follows:

Twice-exceptional learners are students who demonstrate the potential for high achievement or creative productivity in one or more domains such as math, science, technology, the social arts, the visual, spatial, or performing arts or other areas of human productivity AND who manifest one or more disabilities as defined by federal or state eligibility criteria (Reis et al., 2014, p. 222).

They also stated that these high abilities and disabilities together generate a “unique” group of students who may fail to demonstrate either high performance or specific disabilities. They end the first section of their definition with the inclusion of the concept of masking, stating “Their gifts may mask their disabilities and their disabilities may mask their gifts” (p. 222).

Due to G/LD students demonstrating such high ability, their academic achievements might not be as low as that of average ability LD students (McCoach et al., 2001). Brody and Mills (1997) report on three subgroups which can be identified among the G/LD population whose twice-exceptionality can go unnoticed. The first group consists of students identified with LD, but whose disabilities mask their high potentials thus leaving their giftedness unrecognized. Due to inadequate assessment or the possibility that their disability leads to lower Full Scale IQ scores, these students' giftedness often remains hidden. In the second group, students are identified as being gifted, yet their disabilities are unnoticed because their high intelligence masks their learning problems. They often underachieve and have difficulties in school, but these problems may be attributed to factors such as a lack of motivation or poor self-concept. The third group includes students whose giftedness and learning disabilities mask each other, resulting in both exceptionalities remaining unidentified. They typically display average achievement in school and are usually perceived as having average ability.

Although the masking hypothesis is widely accepted in the G/LD field (Gunderson et al., 1987; Silverman, 1989; Waldron and Saphire, 1990; Robinson, 1999), it has also received criticism. McCoach et al. (2001) formulates theoretical and practical problems pertaining to the masking concept. Theoretically, the question can arise whether it is justifiable for average performing students to be referred to as learning disabled and therefore qualify for special services (for a critical discussion, see Gordon et al., 1999). Indeed, Lovett and Sparks (2010, 2011) found that most G/LD identified students have lower IQ scores than typical gifted students and demonstrate higher levels of achievement than typical learning-disabled students, concluding that these students often do not meet traditional criteria for either exceptionality. From a pragmatic approach, this shows clearly that identification of G/LD students is extremely difficult. According to McCoach et al. (2001), screening all average performing students for hidden learning disabilities would not be feasible. Instead, they suggest focusing on the identifying characteristics of G/LD students, so that they can be identified by practitioners before these students begin critically underachieving. Accordingly, despite growing knowledge and insight into this field, identification problems are still ever-present (Reis et al., 2014).

The aforementioned identification issues leave traces in the developmental patterns of G/LD students. Brody and Mills (1997) express concern about the socio-emotional consequences of unrecognized exceptionalities, which can be quite severe. As their exceptionalities are hidden, they do not receive the necessary support to develop well (Gardynik and McDonald, 2005). In school, they face numerous challenges and are often characterized by their seemingly paradoxical needs (Gardynik and McDonald, 2005; Reis et al., 2014). On the one hand, they should be enabled and encouraged to explore their talents, while on the other, they require support with managing their learning difficulties. This ongoing conflict between high potential and disabling weaknesses is confusing for teachers and makes it difficult for the students to form a stable and realistic self-concept (King, 2005). A pattern of recurrent failure often results in a poor academic self-concept, low confidence and increasing frustration with school and with themselves (Weill, 1987; Swesson, 1994; Gardynik and McDonald, 2005; King, 2005; Yssel et al., 2010). They also tend to feel isolated from peers as they do not always fit in with gifted peers, or with students who only have learning disabilities (Yssel et al., 2010). Dole (2000) notes that these characteristics considerably increase the vulnerability of this population, making the recognition and support of non-cognitive characteristics vitally important to G/LD students' learning processes, school and life outcomes. Taken cumulatively, there is a vital need to deliberately and systematically deepen our understanding of the non-cognitive characteristics of G/LD students.

### Research questions

To develop a more in-depth understanding of the characteristics of G/LD students, the following research question and sub-questions were formulated: What are the non-cognitive characteristics of G/LD students, do they relate to relevant contrasting groups of giftedness, and if so, how? The sub-questions are as follows:

1. What “non-cognitive” characteristics can be identified among G/LD students?
2. Are there differences and/or similarities in non-cognitive characteristics of G/LD students compared to non-cognitive characteristics of relevant contrasting groups such as gifted non-disabled students, non-gifted learning-disabled students, and average performing students?

## Methods

### Search procedure

We searched the ERIC, PsycINFO, Medline, and SocINDEX electronic databases for relevant studies in January/February 2017. The following keyword descriptors were used in combination: (“Gifted^*^” or “talent^*^” or “genius” or “intelligen^*^” or “high ability” or “highly able” or “high IQ” or “twice exceptional^*^” or “dual exceptional^*^” or “2e students” or “dual differentiation”) and (“learning disab^*^” or “learning difficult^*^” or “learning problem^*^” or “SLD” or “Dyslex^*^” or “Dyscalculia”). The term “gifted/learning disab^*^” was also used. All the above terms were used in searches of whole texts of publications. However, the term “intelligen^*^” was only applied to searches of “titles,” in order to limit irrelevant hits. The search was systematically conducted on journal articles in academic peer-reviewed journals, and was not limited by year. This process yielded a total of 1,481 hits.

### Inclusion criteria

When assessing excellence in the “intellectual” area of gifted students the most commonly used threshold is an IQ score of 130 (Vaivre-Douret, 2011). In identifying twice-exceptional students, however, this threshold is often lowered to 120 to account for measurement error (Silverman, 1989; Brody and Mills, 1997; Goldstein et al., 2008; Burger-Veltmeijer et al., 2011). In this study, we used a cut-off score of 120 on any composite IQ test to allow for students who are gifted in, e.g., either the verbal or performance area of intelligence. Due to the lack of rigorous empirical data, we decided not to impose restrictions on the learning disabilities part.

### Study selection

To select relevant studies, the list of publications was first filtered by simply reading the titles and abstracts to determine whether they addressed the topic of G/LD students. This procedure resulted in the exclusion of 1,260 studies. During the second filtering, the remaining 221 studies were screened and analyzed on the exclusion criteria shown in Table 1.

**Table 1:** Number of rejected studies based on the selection criteria (second filtering).

**Exclusion criteria**	***n***
No empirical data	64
Did not discuss any non-cognitive characteristics as specified in the present study	65
Did not focus on (intellectually) gifted students with learning disabilities/problems	28
Did not meet inclusion criteria of giftedness as specified in the present study	19
Important methodological information missing	8
Comorbid disorders in participants	6
Total	190

Although most studies did not meet at least one criterion, some studies were excluded on the basis of more than one exclusion criterion. Of the 221 studies, eight were untraceable (i.e., not found either online, through the library or by email to the author). This process resulted in 23 relevant studies.

Table 2 presents descriptive information about the studies included in this review, describing the aim of the study, the age, educational stage, and gender of the selected target group, the type or types of learning disabilities or problems, and the giftedness and learning disabilities criteria used to identify G/LD students. The studies are grouped according to group design studies and individual or multiple case studies. Because some of these studies include subjects who do not meet the selection criteria as formulated above, only partial information from those articles is used. This means that only the subjects (and corresponding information) who are members of the group under study are displayed. Note that the study by Assouline et al. (2010) included both a case study and group design.

**Table 2:** Descriptive summary of the selected empirical studies which at least implicitly discuss characteristics of G/LD students.

**Authors**	**Aim of study**	**n + age/educational stage + gender of selected G/LD cases**	**Type(s) of learning disability/area of difficulty of selected G/LD cases**	**Giftedness criteria for G/LD cases**	**LD criteria for G/LD cases**
**GROUP DESIGNS**					
Assouline et al., 2010	Study including group data and a case study, to determine the effectiveness of cognitive, academic and psychological profiles of G/LD students in understanding their needs	*n* = 14 students 11 boys, 3 girls Age range 8–17	SLD in written expression (*n* = 14); co-occurring SLD in reading (*n* = 5)	General or specific IQ score ≥ 120 on WJIII, WISC-IV or WAIS-III	Standard score on one or more measures of written language at least 1 *SD* below IQ score
Baum and Owen, 1988	Study investigating whether G/LD students differ from G and LD students regarding cognitive and motivational characteristics	*n* = 24 G/LD students *n* = 54 G students *n* = 34 LD students Grades 4–6	No info	VIQ or PIQ > 119 on WISC-R	IQ-achievement discrepancy based on standardized achievement test data and IQ scores
Coleman, 1992	Study investigating whether G/LD students differ from LD students in their use of coping strategies to deal with difficult school situations	*n* = 21 middle school boys in both groups (total *n* = 42) Grades 6–9	No info	VIQ, PIQ, or FSIQ > 125 on WISC-R	Identified as LD by state guidelines: IQ-achievement discrepancy ≥15 as a result of a deficit in underlying psychological processes; alternative explanations ruled out
Hannah and Shore, 1995	Study investigating whether G/LD, G, LD, and average-performing students differ in their use of metacognitive skills and knowledge during a difficult reading task	*n* = 12 male students per group (total *n* = 48) Grades 5–6 and 11–12	No info	VIQ, PIQ, or FSIQ ≥ 127 on WISC-R	Severe IQ-achievement discrepancy in one or more academic areas; alternative explanations ruled out; evident deficits in one or more basic learning processes; academic performance affected to such an extent that special educational services are required
LaFrance, 1995	Study investigating whether creative thinking of G/LD students is similar to that of G and LD students	*n* = 30 students per group (total *n* = 90) Age range 9–14	No info	Any composite IQ score in the 98th percentile on WISC-R	Standardized achievement test scores ≤70th percentile
LaFrance, 1997	Study investigating whether G/LD students differ from G and LD students in cognitive and creative thinking skills, and academic and socio-emotional outcomes	*n* = 30 students per group (total *n* = 90) G/LD group: one female to every 4 or 5 males Age range 9–14	Dyslexia	VIQ, PIQ, or FSIQ 2 SD above average on WISC-R	Meeting criteria for dyslexia (e.g., difficulties in receptive and expressive language; other explanations ruled out; scores on one or more standardized reading/language arts achievement tests <70th percentile
Woodrum and Savage, 1994	Pilot study investigating whether G/LD resemble either G or LD students more in their strength characteristics (i.e., higher cognitive processes, creativity, divergent feelings, and motivation)	*n* = 5 students per group (total *n* = 15) Grades 6-8	No info	VIQ or PIQ ≥ 130 on WISC-R	Severe IQ (VIQ or PIQ)-achievement (reading or mathematics) discrepancy of 1.75 *SD* as determined by a multidisciplinary team
**(MULTIPLE) CASE STUDIES**					
Al-Hroub, 2011	Multiple case study to explore whether multidimensional assessment can be an efficient method for the identification of mathematically gifted LD students	*n* = 5 children 3 boys, 2 girls Age range 9–11	James: spelling and writing difficulties (illegible handwriting) Maria: writing (illegible handwriting) and spelling difficulties William: DCD (dyspraxia), writing difficulties Richard: writing difficulties (illegible handwriting) Anne: written expression difficulties	FSIQ ≥ 120 on WISC-III, alongside documentary evidence, teacher and parent interviews, and dynamic assessment involving a mathematics achievement test	Poor performance on the DST and the Neale Analysis of Reading Ability, alongside documentary evidence, and teacher and parent interviews
Assouline et al., 2006	Two case studies of gifted children with ADHD or SLD, highlighting how students' giftedness and disabilities can be identified through comprehensive assessment	*n* = 1 fifth grade male student	Randy: SLD in written language	No criteria explicitly stated Subject's FSIQ is in the gifted range (98th percentile) on WISC-III	No criteria explicitly stated Analysis of subtest profile indicated LD in subject; achievement test scores “substantiated” SLD
Assouline et al., 2010	Study including group data and a case study, to determine the effectiveness of cognitive, academic and psychological profiles of G/LD students in understanding their needs	*n* = 1 17-year-old male high school student	Disorder of written expression	General or specific IQ score ≥ 120 on WAIS-III	Standard score on one or more measures of written language at least 1 SD below IQ score
Assouline and Whiteman, 2011	Three case studies of gifted children with ADHD, ASD, or SLD, to increase understanding of the twice-exceptionality phenomena and to illustrate complexities of identification and intervention	*n* = 1 fifth grade male student	David: SLD in written language	No criteria explicitly stated Subject's VIQ is within the “very superior” range (99.8th percentile), NVIQ and PS are high average (84th/79th percentiles, respectively) on WISC	No criteria explicitly stated Subject had previously been diagnosed with a SLD; assessment indicated a SLD on the basis of IQ-achievement discrepancy plus RTI
Coleman, 2001	Multiple case study exploring how G/LD students deal with difficult school situations	*n* = 21 middle school boys Grades 6–9	No info	VIQ, PIQ, or FSIQ ≥ 125 on WISC-R	Identified as LD by state guidelines
Cooper et al., 2004	Case study of a spatial-temporal gifted child with dyslexia, providing identification procedures and instructional approaches	*n* = 1 boy K-5	Dyslexia	No criteria explicitly stated Subject's FSIQ = 124, VIQ = 101, and PIQ = 145 on WISC-III	No criteria explicitly stated Subject shows an IQ-achievement discrepancy despite RT
Dare and Nowicki, 2015	Multiple case study examining parent's experiences of their gifted children's identification as twice-exceptional, i.e. LD, ASD, ADHD, or EBD	n = 1 sixth grade girl	Jessica: arithmetic and spelling difficulties	No criteria explicitly stated Subject's FSIQ is within the “very superior” range on the WISC	No criteria explicitly stated Subject's score on achievement testing showed “patterns of weaknesses in arithmetic and spelling that suggested specific learning disabilities”
French, 1982	Case study of a G/LD child highlighting diagnostic and remediation approaches	*n* = 1 9-year-old boy	Considerable difficulty in writing; considerable difficulty in reading, due to a specific problem in visual perception	No criteria explicitly stated Subject's FSIQ is in the ‘superior’ range on the WISC-R	No criteria explicitly stated School documentation of LD, reading 1.5–2 years below grade level
Hannah and Shore, 2008	Multiple case study analyzing G/LD students' use of metacognitive skills during a difficult reading comprehension task	*n* = 12 boys 6 fifth and sixth grade students; 6 eleventh and twelfth grade students	Quentin: reading disability Eugene: reading disability Others: unknown	FSIQ ≥130 on WISC-R or SB; high achievement in at least one academic area based on an individual standardized achievement test or scholastic performance	IQ-achievement discrepancy of ≥1.75 *SD*; alternative explanations ruled out; demonstrable deficits in one or more basic learning processes; academic performance affected to such an extent that special educational services are required
Hua, 2002	Case study exploring the career self-efficacy of a G/LD student	*n* = 1 male high school student	Severe sensory-motor integration problem (resulting in writing difficulties)	Previously identified as gifted and talented subject has an IQ of 135	Previously identified as LD and received special education services based on IEP or 504 plan
McGuire and Yewchuk, 1996	Multiple case study examining G/LD students' use of metacognitive strategies during a reading comprehension think-aloud task	*n* = 4 elementary school students, 3 boys, 1 girl Age range 10–12	Reading difficulties	VIQ or PIQ ≥125 on WISC-R	“Identified by the schools as learning disabled on the basis of learning or behavioral difficulties”; standardized reading achievement scores at least 1 year below grade placement on the reading subtest of the CTBS
Montague, 1991	Multiple case study examining G and G/LD students' use of cognitive and metacognitive strategies during mathematical problem-solving	*n* = 3 G/LD boys, aged 14–16 *n* = 3 G students, 2 girls, 1 boy aged 13–14	No info	IQ 2 or more SD above mean on an individually administered standardized IQ test; showed majority of G characteristics according to a standard checklist	“A disorder in one or more of the basic psychological processes” (i.e., visual, auditory, or language); significant IQ-achievement discrepancy; alternative explanations for LD ruled out
Reis and Colbert, 2004	Multiple case study exploring G/LD students' perceptions about their social and emotional school experiences	*n* = 15 college or university students 9 males and 6 females	No info	Documentary material including information about IQ and/or achievement tests, outstanding performance in one or more academic areas, teacher nomination, and product information from an academic portfolio. All subjects had an VIQ or PIQ ≥125 on WAIS-R	Documentary material including identification during elementary or secondary school and testing information and screening by university staff
Reis et al., 2000	Multiple case study exploring the perceptions of G/LD university students on their use of compensation strategies which facilitate success in an academic setting	*n* = 10 university students 8 males, 2 females (exclusion of Diane and Mike)	Variety of LDs	IQ scores, achievement, and other indicators of performance indicating G; academic success in university setting All subjects had an VIQ, PIQ, or FSIQ ≥124 on WAIS-R	Documentation on identification of student as having a LD
Vespi and Yewchuk, 1992	Multiple case study exploring the social/emotional development of G/LD students	*n* = 4 boys age range 9–12	Andrew: delayed achievement in reading and language Adam: reading and writing difficulties Michael: delayed achievement in reading and language; especially difficulty with written work (poor visual spatial skills) Steven: delayed in reading and language; difficulty in spelling	VIQ, PIQ, or FSIQ ≥120 on WISC-R	Identified academic difficulties and receiving assistance from a special education teacher; a WISC-R VIQ/PIQ discrepancy of ≥18 and/or a subtest scatter of 7, 9, and 10 points between the highest and lowest scaled scores on the Verbal, Performance, or Full Scales, respectively.
Willard-Holt et al., 2013	Mixed-methods study including a survey and multiple case studies, to explore the perspectives of twice-exceptional students [e.g., ASD, LD, AD(H)D, EBD] on learning strategies	*n* = 1 21-year-old male student	Travis: written expression	Identified as gifted by school criteria, i.e., teacher nomination; achievement and IQ scores ≥98th percentile	Identified as having one or more disabilities through comprehensive assessment by a registered psychologist, based on DSM-IV and state criteria
Wormald et al., 2015	Case study exploring the roles of student, parents, and the education system in making optimal development of a G/LD student possible	*n* = 1 male university student	DCD (dyspraxia)	Documentation evidence provided by the parents of the subject Subject's FSIQ is at 98th percentile	Documentation evidence provided by the parents of the subject

*AD(H)D, Attention Deficit (Hyperactivity) Disorder; ASD, Autism Spectrum Disorder; CTBS, Canadian Tests of Basic Skills; DCD, Developmental Coordination Disorder; DSM-IV, Diagnostic and Statistical Manual of Mental Disorders 4th edition; DSM-IV-TR, Diagnostic and Statistical Manual of Mental Disorders 4th edition-Text Revision; DST, Dyslexia Screening Test; EBD, Emotional/Behavioral Disorder; FSIQ, Full-scale IQ; G, Gifted(ness); G/LD, Gifted/Learning Disabled; K-5, Kindergarten-through-5th grade; LD, Learning Disabled/Disability; NVIQ, Non Verbal IQ; PIQ, Performance/Perceptual IQ; PS, Processing Speed factor; RT, Remedial Teaching; RTI, Response to Intervention; SB, Stanford-Binet Intelligence Scales; SD, Standard Deviation; SLD, Specific Learning Disability; VIQ, Verbal IQ; WAIS-III, Wechsler Adults Intelligence Scale 3rd edition; WAIS-R, Wechsler Adults Intelligence Scale-Revised; WISC-III, Wechsler Intelligence Scale for Children 3rd edition; WISC-IV, Wechsler Intelligence Scale for Children 4th edition; WISC-R, Wechsler Intelligence Scale for Children-Revised; WJIII, Woodcock-Johnson 3rd edition*.

The descriptive information in Table 2, which presents the gender of the G/LD students included in the studies, reveals that the vast majority of students are boys. This is in line with the fact that children diagnosed with learning disabilities are predominantly boys (Morrison and Cosden, 1997). When considered from the perspective of the specific difficulty experienced, most are language-related disorders, either in written expression, reading or spelling. Another frequently occurring learning difficulty pertains to the area of (sensory-) motor functioning (e.g., Developmental Coordination Disorder, dyspraxia), often reflected in writing difficulty. Arithmetic difficulties were reported in only one case. The students are diverse in age and educational stage, ranging from elementary school to middle school and college or university. Lastly, Table 2 shows that the studies vary in the learning disability criteria they use to include students. Most studies, however, based the identification of learning disabilities predominantly on the presence of an IQ-achievement discrepancy, although the specific procedures for identifying learning disabilities were not always made explicit. It is apparent that many studies do not require G/LD students to exhibit absolute low achievement in an academic area, in that they are not substantially below average compared to their age-related peers.

An inductive, bottom-up approach was used in identifying the non-cognitive characteristics of G/LD students, in which the perspectives of the authors, parents, teachers, and G/LD students were taken into consideration. This approach was deliberately chosen to allow equal consideration of every kind of non-cognitive characteristic mentioned, and to allow for a complete and in-depth overview. To prevent inaccurate interpretations, characteristics were included using their exact wordings, or as exact as practical, to prevent inaccurate interpretations. In clustering the characteristics, however, some wordings were slightly altered to allow them to be merged into one comprehensive family. From an epistemological viewpoint, the characteristics were content-wise grouped either from the framework of the studies themselves, or inspired by the existing literature on non-cognitive characteristics (Heckman et al., 2006; Farrington et al., 2012; Gutman and Schoon, 2013; García, 2014; Kautz et al., 2014), resulting in 11 comprehensive clusters of behaviors, skills, attitudes, and emotions with which G/LD students can be described. The wording and grouping of the characteristics was conducted by the first author and independently carried out by a second reviewer. Full agreement was achieved among author and reviewer after in-depth discussions on a few characteristics. Final classification was achieved by making use of either contemporary scientific literature (and likewise classifications) or the umbrella category “overall perceptions and misperceptions.”

## Results

The results are divided into two sections, corresponding to the research sub-questions stated in section Research Questions. The first section on non-cognitive characteristics aims to answer the first research question by describing the patterns within and between clusters (i.e., between grouped characteristics) and between the individual characteristics per cluster (i.e., on individual characteristics' level). In the second section comparisons are made between contrasting groups.

### Non-cognitive characteristics

Table 3 displays the 80 non-cognitive characteristics of G/LD students found in the 23 publications. As shown in the first column of Table 3, the following clusters of non-cognitive characteristics were identified: “externalizing problems,” “self-perceptions,” “interpersonal relationships,” “creativity,” “attitudes,” “personality traits,” “emotions,” “resilience and coping,” “motivation,” “metacognition,” and “overall perceptions and misperceptions.” In most clusters, a distinction is made between “positive” and “negative” characteristics, represented by a plus (+) and a minus (–) symbol, respectively. The former refers to characteristics in that cluster which indicate high ability, skills or (socially) desirable outcomes. The latter refers to the characteristics which indicate negative issues, difficulties, or unpleasant and usually undesirable (for themselves or others) feelings and behaviors.

**Table 3:** Non-cognitive characteristics per publication divided into clusters.

**23 publications**→		**(Al-Hroub, 2011)**	**(Assouline et al., 2006)**	**(Assouline et al., 2010)**	**(Assouline and Whiteman, 2011)**	**(Baum and Owen, 1988)**	**(Coleman, 1992)**	**(Coleman, 2001)**	**(Cooper et al., 2004)**	**(Dare and Nowicki, 2015)**	**(French, 1982)**	**(Hannah and Shore, 1995)**	**(Hannah and Shore, 2008)**	**(Hua, 2002)**	**(LaFrance, 1995)**	**(LaFrance, 1997)**	**(McGuire and Yewchuk, 1996)**	**(Montague, 1991)**	**(Reis and Colbert, 2004)**	**(Reis et al., 2000)**	**(Vespi and Yewchuk, 1992)**	**(Willard-Holt et al., 2013)**	**(Woodrum and Savage, 1994)**	**(Wormald et al., 2015)**	**Totals**
**80 Characteristics grouped into 11 comprehensive clusters** ↓																									
**EXTERNALIZING PROBLEMS**																									
Acting out, disruptive/odd behaviors		**I**		**I**		**C, D**								**I**					**C**		**C**			**I**	**7**
Hyperactivity				**C**																					**1**
**SELF-PERCEPTIONS**																									
+	Positive self-concept			**I**					**I**					**I**							**C**				**4**
	Confident (about own abilities)/high self-efficacy								**I**				**C**	**I**						**C**	**C**				**5**
	Self-acceptance							**C**											**C**						**2**
−	Negative self-concept		**I**	**I**		**C**					**I**								**C**		**I**				**6**
	Low academic self-concept					**C**			**I**				**I**	**I**							**C**				**5**
	Lack of confidence/low self-efficacy	**I**				**C, D**							**C**	**I**					**C**		**C**			**I**	**7**
	Feeling “stupid”/“dumb,” perceiving oneself as a failure					**C**		**I**											**C**						**3**
Fluctuating self-concept, puzzled by discrepancy									**I**					**I**					**C**		**C**				**4**
Unrealistic self-expectations																			**C**		**C**				**2**
**INTERPERSONAL RELATIONSHIPS**																									
+	Close relationships with/supported by relatives	**I**	**I**					**I**	**I**	**I**				**I**					**I**		**C**			**I**	**9**
	Close relationships with/supported by teachers/educators/mentor							**C**	**I**					**I**					**I**		**C**			**I**	**6**
	Good social skills										**I**										**C**				**2**
	Popular in class/getting along well with peers	**I**							**I**											**C**				**I**	**4**
−	Conflicts with/misunderstood by parents							**C**						**I**							**I**				**3**
	Negative incidents with teachers/negative teacher interaction													**I**					**C**		**I**	**I**		**I**	**5**
	Misunderstood/underestimated by teachers		**I**											**I**					**C**					**I**	**4**
	No friends in class/not being accepted into a group	**I**																	**C**		**C**				**3**
	Feeling different/distant from peers								**I**	**I**									**I**						**3**
	Issues with peers								**I**	**I**									**C**		**C**				**4**
	Feeling uncomfortable with accommodations in class (because of perceived stigma)									**I**										**I**					**2**
	Socially inappropriate behavior (verbally abusive, bullying)	**I**																	**I**					**I**	**3**
	Difficulties with pragmatic language, difficulty understanding peers	**I**																							**1**
	Social withdrawal, quiet/shyness	**I**		**C**		**C, D**			**I**					**I**					**I**					**I**	**7**
	Being bullied																		**C**					**I**	**2**
**CREATIVITY**																									
+	Highly creative, good creative thinking skills					**C, D**					**I**				**C**	**C**					**C**		**C**		**6**
	Good/creative problem-solving skills	**I**					**C**			**I**			**I**			**C**			**C**						**6**
	Vivid imagination								**I**						**C**								**C**		**3**
	Difficulty with problem-solving																	**C**							**1**
**ATTITUDES**																									
+	Positive attitude toward area of difficulty	**I**																							**1**
−	Negative attitude/feelings toward school/area of difficulty					**C**		**I**					**I**	**I**					**C**	**C**	**C**			**I**	**8**
	Unsure about place in academic setting		**I**																						**1**
**PERSONALITY TRAITS**																									
Easy temperament		**I**							**I**												**C**				**3**
Irritable, envious/scornful		**I**																							**1**
Reflective, thoughtful										**I**				**I**						**C**	**C**				**4**
Expressing sense of humor								**C**							**C**	**C**									**3**
Well-adjusted		**I**		**I**	**I**				**I**												**C**			**I**	**6**
Perfectionism																			**C**						**1**
Independent/mature, striving toward self-sufficiency		**I**						**I**											**I**	**I**		**I**		**I**	**6**
Highly curious, eager to learn		**I**								**I**	**I**											**C**	**C**	**I**	**6**
Fast learner/quick-witted		**I**																						**I**	**2**
Internal locus of control						**C, D**	**C**						**C**			**C**				**C**	**C**				**6**
External locus of control						**C**															**I**				**2**
**EMOTIONS**																									
+	Passionate/enthusiastic (about area of interest)	**G**			**I**					**I**	**I**			**I**							**C**				**6**
	Proud of talents													**I**											**1**
−	Emotional issues/instability			**I**						**I**									**C**	**C**	**C**			**I**	**6**
	Frustration	**I**				**C**	**C**	**C**		**I**	**I**		**I**	**I**					**C**	**I**	**C**	**C**		**I**	**13**
	Anger							**I**						**I**					**C**		**I**				**4**
	Quickly bored	**I**												**I**							**C**				**3**
	Stressed/worried, being tense	**I**		**I**				**C**						**I**							**C**			**I**	**6**
	Fear of failure	**I**						**I**		**I**	**I**								**C**		**C**			**I**	**7**
	Helplessness/feeling overwhelmed	**I**																	**C**		**I**			**I**	**4**
	Embarrassed/self-conscious																		**C**					**I**	**2**
	Feeling weak/tired																			**C**		**I**		**I**	**3**
	Depressed																		**I**		**I**			**I**	**3**
	Feeling traumatized																		**C**					**I**	**2**
	Suicidal thoughts	**I**																	**I**					**I**	**3**
**RESILIENCE AND COPING**																									
+	Great perseverance						**C**				**I**		**I**	**I**				**C**		**C**	**C**			**I**	**8**
	Working (unusually) hard				**I**					**I**	**I**									**C**				**I**	**5**
	Using compensatory techniques							**C**			**I**		**I**	**I**			**I**			**C**	**C**	**I**		**I**	**9**
	Using a variety of coping strategies						**C, D**	**C**	**I**								**C**			**C**				**I**	**6**
	Relying on social support						**C**							**I**							**C**				**3**
	Engagement with out-of-school activities													**I**							**I**				**2**
	School/task avoidance behavior					**C**								**I**					**C**		**C**			**I**	**5**
	Easily quitting/inability to persevere	**I**																**I**	**C**						**3**
	Difficulty with implementing coping strategies																**C**								**1**
	Difficulty completing tasks								**I**																**1**
**MOTIVATION**																									
+	Highly (intrinsically) motivated	**I**							**I**		**I**									**C**	**C**	**C**	**C**		**7**
	High aspirations			**I**					**I**		**I**			**I**							**C**				**5**
	Taking risks, showing initiative													**I**						**C**			**C**		**3**
−	Difficult to motivate/lack of motivation	**I**																	**C**						**2**
**METACOGNITION**																									
+	Having metacognitive knowledge											**C, D**						**C**							**2**
	Displaying good metacognitive skills											**C, D**	**C**				**C**			**C**					**4**
	Using self-regulation strategies						**C**						**C**	**C**							**C**				**4**
	Self-awareness (of strengths and weaknesses, about how to optimize own learning)								**I**				**C**	**I**					**C**	**C**	**C**	**I**		**I**	**8**
−	Metacognitive difficulties																**C**	**C**							**2**
	Difficulty organizing work/poor study habits	**I**						**I**						**I**							**I**			**I**	**5**
**OVERALL PERCEPTIONS AND MISPERCEPTIONS**																									
Perceived as “lazy” by teachers/parents			**I**																**C**	**C**					**3**
Perceived as developmentally delayed														**I**					**C**					**I**	**3**
Totals		**27**	**5**	**9**	**3**	**12**	**7**	**15**	**17**	**13**	**12**	**2**	**12**	**31**	**3**	**4**	**5**	**5**	**36**	**19**	**41**	**8**	**5**	**33**	**324**

*C, common characteristic; D, significant distinguishing characteristic from at least one contrast group; I, individual characteristic*.

The next columns in Table 3 identify the publications in which the characteristics were mentioned. The publications displayed in bold are group design studies. “I” denotes “individual” characteristics, applying to individual cases often mentioned in (multiple) case studies, whereas “C” denotes “common” characteristics which apply to all or most of the cases in a study (both group design and multiple case studies). The number of Is and Cs seem to be evenly distributed across the different clusters, with the exception of the metacognition cluster which shows predominantly common characteristics. Lastly, “D” denotes “distinguishing” characteristics, meaning that G/LD students significantly differ from at least one contrasting group.

The last column of Table 3 shows the total number of publications in which each characteristic was mentioned, to be discussed in detail in section Characteristics Per Cluster. The bottom row gives the total number of characteristics mentioned by each publication. This shows that the five publications with the highest number of marked characteristics (>20) are individual or multiple case studies (Vespi and Yewchuk, 1992; Hua, 2002; Reis and Colbert, 2004; Al-Hroub, 2011; Wormald et al., 2015).

#### Patterns within and between clusters

It can be readily observed that G/LD students show a pronounced duality when the 11 comprehensive clusters of non-cognitive characteristics are analyzed. It is notable that there are great contrasts *between* different clusters, as well as contrasts *within* clusters. When looking at contrasts between clusters, the G/LD students show high levels of negative emotions, negative attitudes, low self-perceptions and adverse interpersonal relationships. However, they also exhibit high levels of motivation, great resilience and coping skills, and possess positive personality traits. Table 3 also shows that many studies report that G/LD students exhibit externalizing problems. Given their high levels of negative emotions and social withdrawal, the results also indicate the presence of vast internalizing problems.

Much within-cluster duality is noticeable when each cluster is considered individually. Contrasts are especially pronounced within the cluster of self-perceptions, in which positive self-concept and high self-efficacy appear alongside negative self-concept and lack of confidence. Contrasts are also evident in the resilience and coping cluster, in which the use of coping strategies, great perseverance and hard work concur with school or task avoidance behavior. Lastly, contrasts are present in the metacognition cluster, in which good metacognitive skills as well as poor study habits are co-represented.

An important note to these findings is that the foregoing dualities may be either inter or intra-individual duality/variability, or both. It is not always clear if the contrasts are differences *between* individuals and/or if they exist *within* individuals. However, when analyzing marked characteristics per publication, a high level of intra-individual variability/duality in the subjects can be observed, especially in the case studies from Assouline et al. (2010), Hua (2002), and Wormald et al. (2015). The same subjects show pronounced dualities both across different clusters and within clusters. This issue is addressed below in greater detail.

#### Characteristics per cluster

The first cluster in Table 3, “externalizing problems,” shows that multiple studies (*n* = 7) report that G/LD students exhibit acting out behavior or odd and disruptive behavior. The high number of instances could be related to the high number of boys included in the studies in this review (see section Study Selection), since these behavioral problems are mostly prevalent among boys (Weis, 2014). Although this characteristic seems to occur frequently within this population, the study by Assouline et al. (2010), comprising 14 G/LD students, showed that there were substantial individual differences in the degree of their externalizing problems (i.e., inter-individual duality). Some students had clinically significant scores in this area, whereas others did not, yet teachers reported more odd behaviors in these students compared to typically developing students of their age. Owing to the Assouline et al. (2010) study's sample size being too small to allow for statistical analysis, it is unclear whether these results are representative of the G/LD population as a whole.

The “self-perceptions” cluster, notable for its pronounced contrasts, shows a high number of marks in both negative and positive area. Characteristics such as a negative (academic) self-concept (*n* = 5+6) and a lack of confidence (*n* = 7) seem slightly predominant over positive characteristics such as positive self-concept (*n* = 4) and high confidence (*n* = 5). Dare and Nowicki (2015) reported that their subject experienced anxiety, frustration, and lowered self-esteem in her area of difficulty, and that this relative weakness had a major impact on her psychological wellbeing. Additionally, French (1982) mentioned in her case study that this negative view is reinforced when the subject compared his performance to that of his peers. However, it is evident that not all G/LD students have low self-perceptions; indeed, they may even have a high degree of confidence and positive images about themselves (i.e., inter-individual duality). It seems that when their social environment allows for high expectations and provides encouragement and emotional support, G/LD students can gain a positive self-concept (Vespi and Yewchuk, 1992; Hua, 2002; Cooper et al., 2004). This may suggest that there is also intra-individual variability related to the support received from the social environment. Beyond and above, studies also mentioned that G/LD students have fluctuating self-concepts and are puzzled by the discrepancy between their high potential and disabling weaknesses (*n* = 4). Several authors reported that their subjects found it difficult to reconcile their high capabilities with their low performance and failure at school, resulting in inconsistencies in self-perceptions and self-images which tended to fluctuate over time (Vespi and Yewchuk, 1992; Hua, 2002; Reis and Colbert, 2004). These findings are in line with the aforementioned findings in the field of G/LD research (Swesson, 1994; King, 2005; Yssel et al., 2010).

The cluster of “interpersonal relationships” also shows a pronounced duality, especially when it comes to the often-mentioned negative incidents with teachers (*n* = 5) and being misunderstood by teachers (*n* = 4), while at the same time also having close and supportive relationships with teachers (*n* = 6). Several authors described that their subjects had very positive relationships with teachers who focused on their talents rather than their weaknesses, gave them (learning) opportunities, and provided them with emotional connections and encouragement to feel comfortable at school (Vespi and Yewchuk, 1992; Hua, 2002; Cooper et al., 2004). However, several studies mention that G/LD students also had very painful memories and negative experiences. For example, teachers being unwilling to accommodate their needs (Hua, 2002), punishing them for not finishing tasks on time and denying them opportunities to use compensation strategies (Reis and Colbert, 2004). Intra-individual duality can also be found in relation to their peers. For example, in their multiple case studies, Vespi and Yewchuk (1992) reveal that G/LD students generally had positive social skills but did not always use them. Therefore, although they seem to know how to make and keep friends, their socials skills are often weak in terms of the relationships they hold with their peers. Another notable finding within the cluster of interpersonal relationships concerns the commonly cited close and supportive relationships with relatives (*n* = 9). Many G/LD students experienced strong parental advocacy (Cooper et al., 2004; Dare and Nowicki, 2015), with their parents seeking out additional assistance beyond the educational system because of their children's frustration with school (Dare and Nowicki, 2015). A final notable finding concerns the often-mentioned characteristic of social withdrawal (*n* = 7). Wormald et al. (2015) mention that for their subject, the mental and physical strain of dealing with his difficulties and frustration with school burdened his ability to maintain close social relationships, which led him to withdraw socially and isolate himself.

Within the “creativity” group the results differ less. Considering the frequency with which characteristics such as creative thinking (*n* = 6) and creative problem-solving skills (*n* = 6) are mentioned, G/LD students seem to take creative approaches to their tasks. LaFrance (1997) argued that G/LD students exhibit creative strengths particularly in problem-solving, synthesizing dissimilar concepts, and the intuitive aspects of creative thinking.

In the “attitudes” cluster it is clear that G/LD students generally show a negative attitude toward school and their area of difficulty (*n* = 8). Vespi and Yewchuk (1992) noted that the subjects in their study often showed negative attitudes toward school and displayed negative approaches and emotions to academic tasks. The students reported that they often felt bored at school due to repetitive tasks and their frustration with difficult tasks.

The cluster of “personality traits” shows primarily “positive” characteristics. The results show that G/LD students tend to be well-adjusted (*n* = 6), highly curious (*n* = 6), and independent and striving toward self-sufficiency (*n* = 6). Cooper et al. (2004) conclude that the profiles of G/LD students should not always be linked with behavioral problems or inattention. In fact, in his case study, the subject was well able to adapt to situations and his attention remained focused at school. This G/LD student, however, had a supportive family who advocated for him, understanding teachers, and his academic needs were fulfilled, which probably greatly supported his overall well-adjusted behavior. This finding may indicate that the inter-individual duality between well-adjusted behavior and the externalizing behavior problems of G/LD students could be the result of differences in the level of support these students receive from their social environments. Other studies reported high levels of curiosity and eagerness to learn in their subjects, despite the vastness of strong negative feelings and stressors experienced in their academic situation (French, 1982; Wormald et al., 2015). Another finding shows that G/LD students tend to exhibit an internal locus of control (*n* = 6), in that they see themselves as responsible for their own successes and failures (Vespi and Yewchuk, 1992; Reis et al., 2000). Additionally, Reis et al. (2000) found that G/LD students attributed their school success to their ability to use compensation strategies, hard work and an appropriate attitude.

In the “emotions” cluster, negative emotions clearly stand out the most, as shown by the wide range of negative emotional states. A substantial number of authors mention fear of failure (*n* = 7), emotional issues or instability (*n* = 6), feelings of stress and tension (*n* = 6), and most frequently, feelings of frustration (*n* = 13). These high states of negative emotions somewhat oppose what might be expected considering the overrepresentation of boys in these studies and given the fact that (test) anxiety, depression, suicidal thoughts, and other emotional issues are shown to be more prevalent among girls (Weis, 2014). However, the findings are in line with research by Nicpon et al. (2011), who found G/LD students' negative emotionality to be the only distinctive psychosocial factor. Several authors note that some students were so affected by negative school experiences resulting from the discrepancy between their high potential and learning disabilities that they sought professional counseling (Reis et al., 2000; Reis and Colbert, 2004). Unfortunately, for some students this discrepancy and painful experiences led them to feelings of depression, trauma and suicidal thoughts (Reis and Colbert, 2004; Wormald et al., 2015). A sense of frustration also seems to be ever-present within the population of twice-exceptional students. Multiple authors report that for G/LD students, their rather extreme strengths and weaknesses make schooling a very frustrating experience (Vespi and Yewchuk, 1992; Hua, 2002; Dare and Nowicki, 2015). Vespi and Yewchuk (1992) note that G/LD students' awareness of their high capabilities makes it difficult for them to deal with underachievement. Many authors mention that these students express frustration with tears, tension, and self-doubt (Vespi and Yewchuk, 1992; Dare and Nowicki, 2015), or in with acting out and avoidance behavior (Vespi and Yewchuk, 1992; Hua, 2002; Reis and Colbert, 2004). This finding illustrates the inter- and intra-individual duality between internalizing and externalizing problems as a result of frustration. On the positive side, multiple publications mention that G/LD students also have very positive feelings, and feel passionate and enthusiastic about their areas of interest (*n* = 6).

The “resilience and coping” cluster overall shows more “positive” than “negative” characteristics. In fact, within this cluster, there are a few positive characteristics with a large amount of marks, namely “perseverance” (*n* = 8) and “using compensatory techniques” (*n* = 9). Students also tend to “work hard” (*n* = 5) and “use a variety of coping strategies” (*n* = 6). On the other hand, studies also reported that G/LD students display “school or task avoidance behavior” (*n* = 5) and are “unable to persevere” (*n* = 3). According to the student's mother in the Wormald et al. (2015) case study, the student exhibited exceptional perseverance, which he developed due to his learning disability and his love for learning. Montague (1991) also observed that despite the subjects' struggles in solving problems, they persevered and demonstrated an outstanding level of tolerance. On the part of compensatory strategies, Reis et al. (2000) explored the compensatory techniques reported to be used by G/LD students in order to be successful in the academic setting. They revealed that these students compensate for their learning difficulties variously, including study and time management strategies, cognitive/learning strategies (e.g., chunking information), compensatory supports (e.g., use of computers), self-advocacy (e.g., requesting extra help from professors), and the development of an individual, tailored plan (e.g., taking a reduced course load). These results (particularly the use of cognitive/learning strategies) illustrate the concept of masking, in that students use their cognitive strengths to compensate for their learning disabilities, which can result in their disabilities remaining unnoticed (Brody and Mills, 1997). Based on interviews with middle school boys, Coleman (2001) found that G/LD students use different coping strategies to deal with frustrating school situations. These included working to stay positive and control anxiety, developing a study system, studying harder, and relying on parental support. In contrast, avoidance behavior was reported to occur concurrently (Vespi and Yewchuk, 1992; Wormald et al., 2015), emphasizing intra-individual variability. Vespi and Yewchuk (1992) found that most students in their sample tended to avoid school tasks when they anticipated failure, or rushed through the task to finish it as soon as possible. Likewise, Wormald et al. (2015) noted the same pattern in their subject, who reported having developed several avoidance strategies to circumvent situations he sensed to be problematic.

The cluster of “motivation” shows highly positive characteristics. G/LD students were said to be highly (intrinsically) motivated (*n* = 7) and to have high aspirations for the future (*n* = 5). In their case studies, Hua (2002) and Reis et al. (2000) both described that their subjects set high and clear goals for themselves, and were willing to do whatever it took to achieve them. These students realized that despite the numerous challenges and difficulties they faced, they would have to stay at school and work hard to obtain a college or university degree. The primary motivation for these students were their goals, strong belief in their own abilities and an inner need to realize their potential. Only two publications reported G/LD students as lacking motivation or being difficult to motivate (Reis and Colbert, 2004; Al-Hroub, 2011).

The prevalence of characteristics marked as common, especially “positive” characteristics, is notable in the “metacognition” cluster. This means that characteristics of “self-awareness” (*n* = 8), “good metacognitive skills” (*n* = 4), and “using self-regulation strategies” (*n* = 4) were reported in a larger number of G/LD students. Studies also reported that G/LD students experience difficulties organizing their work and have poor study habits (*n* = 5). The most remarkable finding within this cluster is the high occurrence of “self-awareness” marks (*n* = 8). Several authors noted that the G/LD students included in their studies were generally well aware of their high capabilities (Vespi and Yewchuk, 1992; Hua, 2002; Cooper et al., 2004; Reis and Colbert, 2004), which increased their self-confidence (Vespi and Yewchuk, 1992), and of their weaknesses, their needs and their optimal learning rate (Vespi and Yewchuk, 1992; Cooper et al., 2004; Reis and Colbert, 2004; Willard-Holt et al., 2013). Four studies investigated metacognitive skills among G/LD students (Montague, 1991; Hannah and Shore, 1995, 2008; McGuire and Yewchuk, 1996), but these results are less straightforward. McGuire and Yewchuk (1996) and Montague (1991) reported that their subjects had metacognitive strategy knowledge, but were not able to implement these strategies effectively. Montague (1991) adds that the students seemed to be relatively unaware of their strategy knowledge, and had difficulties in coordinating the use of different strategies. On the other hand, Hannah and Shore (2008) reported that many of their subjects actively use metacognitive skills by monitoring, evaluating, and controlling their reading. Additionally, older students tended to monitor their comprehension more actively than younger students. This finding also demonstrates inter-individual duality, seemingly related to variation in age.

The last cluster, “overall perceptions and misperceptions,” shows that G/LD students were reported to be perceived as lazy by their teachers or parents (*n* = 3), and even perceived as being developmentally delayed (*n* = 3). Assouline et al. (2006) describe in their case study the friction between the school's perception of an average, unmotivated student and the mother's perception of her gifted and unchallenged child. The student, at that time not yet identified as G/LD, was often perceived as lazy by his elementary school teachers, who did not acknowledge or recognize the signs of his giftedness. In line with these experiences, Reis and Colbert (2004) noted that some of their subjects reported traumatic experiences because they were placed in a self-contained special education class in which most students were developmentally delayed. These misperceptions by teachers about G/LD students might be related to their confusion at how both exceptionalities could concur within the same individual (Silverman, 1989).

Overall, the non-cognitive characteristics which have the highest prevalence among G/LD students (see Table 4) are the “use of compensatory techniques” (9), “close relationships with/supported by relatives” (9), “self-awareness” (8), “strong perseverance” (8), “negative attitude toward school” (8) and—last but not least—“high frustration with school” (13).

**Table 4:** Most common non-cognitive characteristics of G/LD students mentioned in studies involved (>6).

**Non-cognitive characteristics**	**Number of studies (*n*)**
Experiencing frustration	13
Using compensatory techniques	9
Close relationships with/ supported by relatives	9
Negative attitude toward school/area of difficulty	8
Great perseverance	8
Self-awareness	8
Social withdrawal, quiet/shyness	7
Lack of confidence/ low self-efficacy	7
Acting out, disruptive/odd behaviors	7
Fear of failure	7
Highly (intrinsically) motivated	7

### Comparisons with contrasting groups

Comparing G/LD students' non-cognitive characteristics to those of contrasting groups can yield relevant information on whether the G/LD population is a clearly distinguishable group with a unique set of characteristics. Furthermore, it can provide insight into how characteristics from both exceptionalities come together, and more specifically, whether G/LD students resemble their gifted peers more or their learning-disabled peers. In total, seven publications included contrasting groups, of which six contain group data (Baum and Owen, 1988; Coleman, 1992; Woodrum and Savage, 1994; Hannah and Shore, 1995; LaFrance, 1995, 1997) and one is a multiple case study which only compared the groups qualitatively (Montague, 1991). When analyzing the significant distinguishing characteristics (Ds) of G/LD students in Table 3, only a few can be found, scattered across the different clusters. Baum and Owen (1988) found the three different groups of G, G/LD, and LD students to be distinct, in that they significantly differ in attribution style, self-efficacy, creativity and behavior characteristics. In other words, G/LD students show more disruptive behavior and feel shy and less efficacious in school compared to students from the other groups. In their study on metacognitive skills, Hannah and Shore (1995) found significant differences between gifted and G/LD students and between learning disabled and average-performing students: the former exceeded the latter in their metacognitive performance. Lastly, Coleman (1992) revealed that G/LD and LD students significantly differ in the coping strategies they use to deal with difficult school situations. Specifically, G/LD students reported more planned problem solving, while LD students reported more avoidance behavior, distancing, and helplessness.

Overall, these results give indications which point to the uniqueness of the G/LD group. Since evidence is still scarce and scattered across different characteristics and clusters, the results are far from conclusive. Not all the studies found G/LD students to be distinctive in their non-cognitive characteristics, which may partly be due to the small sample sizes of the studies. Regarding similarities with their gifted and learning-disabled peers, the studies are also not quite consistent. Some studies showed that G/LD students tend to resemble their gifted peers more (Woodrum and Savage, 1994; Hannah and Shore, 1995), while others show that they most resemble learning disabled students (Baum and Owen, 1988). Other studies showed mixed results (LaFrance, 1995, 1997).

## Conclusion and discussion

This review performed an in-depth analysis and comprehensive synthesis of the non-cognitive characteristics of G/LD students for identification and intervention purposes. In the following sections the research questions will be answered in the context of the main findings, the limitations of this study will be presented and, finally, practical implications and recommendations will be elaborated on.

### Conclusions

In answer to the first research question regarding the non-cognitive characteristics discernible among G/LD students, it can be concluded that several characteristics are very common among these students. For example, they tend to have low confidence and negative attitudes toward school, they are very self-aware, they show great perseverance and they tend to be socially withdrawn. One characteristic they all seem to have in common is the high degree of frustration they experience from the discrepancy between their high potential and low school performance. This finding is consistent with Coleman (1992), who concludes that great variety can be observed among the G/LD population, but a common feature seems to be their frustration with school.

Another main finding of this review is that G/LD students demonstrate a pronounced duality in their non-cognitive characteristics. This confirms that alongside contrasts between high ability and low academic performance in the cognitive domain, these students can also be characterized as having contrasts in the non-cognitive domain. On the one hand, students show high levels of negative emotions, negative attitudes, low self-perceptions, and adverse interpersonal relationships. On the other hand, they exhibit high levels of motivation, great resilience and coping skills, and possess positive personality traits. These results stress that alongside the often-reported inter-individual variability in G/LD students' characteristics (Brody and Mills, 1997; Reis and McCoach, 2000; Nicpon et al., 2011), students also show a great deal of intra-individual variability/duality. The social environment might explain inter- and intra-individual variability in their non-cognitive characteristics. In line with self-determination theory (Deci and Ryan, 1985), we can assume that these students profit from the support and encouragement provided by parents and teachers, and in case their academic and other needs are met, they might tend to exhibit fewer learning and behavioral problems, have better self-concepts, experience fewer negative emotions and feel more engaged. Far more research is, however, needed to disentangle how positive attitudes of parents actually foster gifted children's motivational development in the home situation (Garn et al., 2010).

When G/LD students are compared with relevant contrasting groups, we can conclude that a few indications point to the G/LD category as a distinct group of students who possess unique characteristics. The results, however, are far too inconsistent to draw firm conclusions.

### Limitations

First, as the process of identifying characteristics is not free of interpretation, it is possible that some characteristics might have been overlooked or improperly identified. The same applies to combining characteristics into clusters, where some characteristics could have been assigned to other clusters. For example, the characteristic “eager to learn” from the “personality traits” cluster could also be considered to belong to the “motivation” cluster.

Another limitation of this study is that no restrictions were placed upon either the inclusion criteria on learning disabilities or the year of publication. Since identification criteria and conceptualizations (due to earlier publications) of learning disabilities were allowed to vary, the focus group of this study is less refined, which might have added to the inter-individual duality between students' non-cognitive characteristics.

A final critical remark concerns the large number of positive characteristics found in this review, such as high motivation, high confidence, great perseverance, and well-adjusted behavior. However, these findings should be understood in view of the fact that most G/LD students included in these studies had already been identified as being twice-exceptional, and that some studies deliberately included G/LD students who were academically successful or who had developed into successful adults. Therefore, the “true” population of G/LD students might show somewhat less encouraging characteristics. More importantly, the very negative emotions, attitudes and self-perceptions experienced by this population of students means that they are in fact very vulnerable.

### Implications and recommendations

Most of the studies involved in this review included G/LD students with language-related learning disabilities or sensory-motor problems, the majority of whom were boys. Therefore, research should explore whether the characteristics found in this review are similar for girls and for students having learning disabilities in other areas (e.g., Non-verbal Learning Disorders, dyscalculia). As regards the inclusion criteria, this study focussed solely on giftedness but further research should also consider stricter identification criteria on the part of learning disabilities (see Lovett and Sparks, 2011).

Further research might explore whether differential effects in the prevalence of non-cognitive characteristics are to be noticed in age-related differences, in the type and severity of learning disabilities, in co-morbidity with other conditions, and in cultural differences embedded within different educational systems across countries. Although this kind of in-depth analysis felt beyond the scope of this contribution, it might shed a more fine-grained view on the generalizability of our findings. The limited (or absence of) information provided within the selected articles, impeded us to conduct this kind of in-depth analysis. If available, professional (school) counselors, who are in an excellent position to serve as agents of change, might profit from these gender-, age-, comorbidity-, and culturally related differences to optimize and accommodate G/LD students in school and in community (Foley-Nicpon and Assouline, 2015).

The very commonly observed high degree of frustration with school of G/LD students might answer the practical problem of identification formulated by McCoach et al. (2001). This pertains to the question of how to distinguish a G/LD student whose performance is average because of masking, from a student with a truly average scoring profile. The findings from this review indicate that the first type of students would experience a high degree of frustration due to their high ability not being expressed in their performance. The second type of student, however, performs more in line with his or her potential, so one could imagine that strong feelings of frustration are not that likely to be present in this type of student. This underlines the practical importance of also considering *non-cognitive* characteristics, in particular feelings of frustration, because these students cannot easily be recognized solely from their cognitive characteristics.

All in all, in aiming to define a decisive profile for G/LD students, both educational practitioners and researchers should systematically work according to the comprehensive list of characteristics set out in Table 3, to explore the patterns of dualities at the inter and intra-individual level in detail. It is therefore vital to look at these students' entire profiles, because these clusters of characteristics are conceptually and empirically interwoven and, in addition, often act in concert. Meanwhile, this list of characteristics could serve as a guideline for needs-based assessment, where the relative and absolute strengths and weaknesses of twice-exceptional students can be identified (see Burger-Veltmeijer et al., 2016). This could prevent occurrences of biased and rather unsystematic assessment practices and offer more fine-grained insights into their sometimes conflicting needs.

To close, since needs-based assessment does not focus on classification but rather on individual profiles and identifying needs, this could also serve students who are noticeably underachieving, but who do not meet the criteria for a G/LD classification. Tailored, fine-grained services could thus be provided to students who evidently struggle in fulfilling their (learning) potential. The overall finding of this systematic review highlights the importance of the social environment to justify the social-emotional and (meta-)cognitive potential of twice-exceptional learners.

## Author contributions

EB launched the review idea of twice-exceptionality (gifted students with learning disabilities), has conducted the systematic review procedure, has extensively analyzed the articles, coded all articles involved, and has written most of the article, though in the format of a thesis. AM initiated the review process and supervised EB in the entire scientific process of conducting a systematic review, providing feedback on the procedure and on the written part of the thesis, and has rewritten parts of the thesis preparatory for publication submission. The process of systematic reviewing was in mindful cooperation between EB and AM.

### Conflict of interest statement

The authors declare that the research was conducted in the absence of any commercial or financial relationships that could be construed as a potential conflict of interest.

## Footnotes

^1^(IDEA, 2004, 602[30][a])
